# Academy of Stomatology

**Published:** 1900-07

**Authors:** 

**Affiliations:** Academy of Stomatology


### Reports of Society Meetings.

### ACADEMY OF STOMATOLOGY.

A REGULAR meeting of the Academy of Stomatology was held on the evening of February 27, 1900, at the rooms of the Academy, 1731 Chestnut Street, Philadelphia, the President, Professor E. C. Kirk, in the chair.

A paper upon the subject of "Hypnotic Suggestion in the Practice of Dentistry" was read by Professor Walter H. Neall, of Philadelphia.

(For Professor Neall's paper, see page 437.)

*The President.*—You have listened to this very interesting paper, and it seems to me a very appropriate subject, one that appeals very definitely to us as dental practitioners. I trust that there will be a full discussion, and I will call upon Dr. Truman, who has some knowledge of this subject, to open the discussion.

*Dr. James Truman.*—The study of this subject is one profoundly interesting, more so, perhaps, in former years than at the present time. I was perhaps seventeen years of age when I first saw a patient subjected to what was then termed "mesmerism." The sight created in me profound astonishment and interest, and from that period up to the present time I have more or less investigated the matter.

It has been one hundred and twenty-five years since Mesmer first broached the idea of the control of the human mind, and many have an idea that Mesmer was what we call a quack. That is a very great mistake. He was a man of superior education, learned in divinity, because he was a theologian at first, and subsequently studied medicine, graduating in Germany, so that he was by no means an empirical individual. Yet he had a great many of the characteristics of what we would term "quackery" about him. It was not, however, until 1844—that remarkable year which developed anæsthesia—that Reichenbach gave forth the idea that there was a force in the human organism, which he termed "odic force," or "odyle." Now, Reichenbach was a distinguished scientific man. He was the discoverer of creosote and paraffin. He thoroughly investigated this force and confirmed Mesmer's idea that there was a certain aura in the human organism. He demonstrated, through a large number of experiments, this odic force. I do not know whether this has been confirmed by scientific observation since his day.

Now, the subject of hypnotism, which was the name originally given by Braid fifty years ago, has been brought up every now and then in dentistry and in medicine, but more especially perhaps as an aid to dental operations. I would not deny that it cannot be made effective. I have seen too much of it to assert that pain cannot be relieved materially by suggestions of this kind. It is a positive force. I have not the slightest doubt about that, but the question arises, "How are we to use it?" Would it be more justifiable to use it in the office without a third person present than in the case of administration of an anæsthetic? Would it not very soon be reported that Dr. So-and-so was using hypnotism? And you know the general feeling abroad in regard to this influence. I am afraid that the practice of a dentist adopting this method would be likely to suffer, and yet that has nothing to do with the main fact after all,—that pain can be prevented. Hypnotism is, to a certain extent, an analgesic, and operations can be performed under it. That I know. My father, who was a medical man, used it to a very great extent in various ways, not only for the extraction of teeth, but in the sick-room. Many people who are in a nervous, hysterical condition imagine themselves sick, and with such that power, properly used, may be of great advantage.

Now, somnambulism has been referred to in the paper. I do not think that there is any difference between the two states. One is self-induced, so to speak. Those who have had any experience with somnambulism know that it is a very peculiar condition. Perhaps I have had the fortune, or misfortune, to have had a great deal to do with it. I had it in one of my family,—a daughter nine years of age, whom we could not control. At night she would get up and walk around the house. We were obliged to fasten down the windows, and she gave us an immense amount of trouble. I had an assistant at that time who boarded with me. That young man would get up in the night and go out into the street. I have known him to go up to my office, start the fire, burn the lights, spend most of the night there, come back, get into bed, and be wholly unconscious of having done anything. I was obliged to use very strong measures with him in order to stop it. That seemed to have the desired effect, but it did not prevent the eternal racket that was continually going on up-stairs.

Now, I think from all that I have observed, and the years of observation that many of our scientists have had, that it is rather late in the day for any one to taboo this hypnotism. I am glad that Dr. Neall has given us a very clear statement of his experience with it. There is a vast deal of benefit which may be derived from it, and it is high time that scientists should take hold of it instead of ignoring the facts surrounding it.

*Dr. M. Schamberg.*—I have investigated this subject and have given it considerable thought and study, but I must say that within the past few years, for somewhat the same reasons that Dr. Truman has advised not to adopt it in practice, I have given it up to a very large degree. I feared the result of criticism in its use in a private office when alone with a patient, and where a patient who does not fully understand the conditions and value of hypnotism might ruin one's reputation.

I want to cite a few experiments that I have conducted along the dental line, and I think they picture somewhat the various classes that the essayist has brought to your notice. One case occurred when I first began dentistry, in the extracting-room of the Chicago College of Dental Surgery, when a patient demanded the use of cocaine for the extraction of a tooth. The demonstrator told me that they did not use the drug, and advised me to try to pacify the patient or induce her to have it taken out by some other means. I finally resorted to one form of suggestion, and taking a small portion of plaster of Paris, I mixed it with a little water on a slab, in a very careful manner, meanwhile telling the patient that cocaine was highly toxic and that in case any of it should reach her tongue she was to spit it out, etc. I then applied very carefully with cotton a small portion of this plaster to the gum about the tooth. I extracted the tooth and the patient claimed that she had absolutely no pain whatever, and even insisted that I take a fee.

Another case is one in which I had my brother put a patient of mine into an hypnotic state. I removed a number of roots from the mouth absolutely without pain. The patient awoke, and would scarcely believe that I had removed them. There are numerous cases of this kind. The unfortunate thing about hypnotism is that it has been the subject of amusement rather than scientific research. Men are travelling about the country exhibiting their powers of hypnotism,—sometimes genuine,—causing their subjects to do ridiculous feats upon the stage. This naturally arouses in the minds of those who are inclined to be sceptical a very peculiar idea about hypnotism and anything but a scientific view of it.

I must say, however, that there is a wide field in hypnotism itself. We all know the immense amount of value that suggestive influence over our patients has in our dental offices. There are exceptional cases, however, in which we deem it advisable to hypnotize a patient; by that I mean to put them in an hypnotic state. While the principles of suggestion and hypnotism are somewhat the same, hypnotism itself means the state of this peculiar sleep, and it has been thoroughly investigated.

Many years back they divided the state of hypnotism into three states,—lethargic, cataleptic, and the somnambulistic states,—and these states carry with them certain phenomena. You must conduct certain experiments under one and certain under another. In the cataleptic state the patient will obey orders and follow out suggestions as they are given by the operator. In the cataleptic state one may become rigid, or many feats of an athletic nature be conducted. In the somnambulistic state we have suggestive conditions occurring, as described by Dr. Truman. Unfortunately it is a condition which is not understood as are other sciences. The subject is vague; we cannot get down to the actual cause of the conditions that hypnotism will produce. They are really so marvellous and perplexing that it is dangerous on that account.

I will relate one peculiar experiment that I was in the custom of conducting while the patient was deeply in the lethargic state. I would state that one side of the body was thoroughly anæsthetized, and that when injuries were inflicted, such as pricking the side of the body, the sensation would be transferred to the opposite side. I would then prick the hand of the patient with a pin, and the patient would evince pain in the opposite hand, showing that anæsthesia was produced on the one side and the sensation imparted to the opposite side. Then there is the post-hypnotic stage, in which patients will follow out suggestions that were made in the hypnotic state, but which may be directed for a period of even a year after the experiments, the patient at that time obeying the will of the operator or the suggestion made while in the hypnotic state.

These things are so perplexing that one is immediately looked upon as a very curious creature who is able to accomplish these peculiar things. Had I prepared remarks for this evening, I would have been able to give you a more valuable talk on the subject, but I have simply given you what few facts have come to my mind since rising to my feet.

*Dr. W. H. Whitslar* (Cleveland).—I am not familiar with the entire matter, but all those who have had some experience in dentistry know that there is a certain amount of hypnotism which we exercise over our patients. There is a certain aura in which we live whilst we are in conjunction with these people in operating for them, and as we control this aura so we control their minds. The subject of simple suggestion is the one with which we desire most to deal. I think the fourth class should be eliminated from practice, because of the dangers into which we may fall by creating unpopular opinions against us. There is one thing which we may do in this work of suggestion, and which we all strive to attain unto, and that is the elimination of fear. When we have eliminated the fear of pain, then we have control over the patient and are able to produce the results that we desire. Then the pain of working upon the ends of the dentinal fibrillæ is lessened. I think that when we are in the state of fear the brain-cells are like the turbulent waters of Niagara as they are passing through the rapids,—our brain-cells are jumping here and there. Where we have subdued the turbulency it produces an equilibrium of nerve-action, and consequently fear is eliminated and pain is lessened.

*Dr. E. C. Kirk.*—The thing that we actually experience in our persons gives us the most definite and solid foundation for belief, and there are certain workings of the human mind that one may study in his own person. From a philosophic point I can conceive of no more wonderful thing in nature than the phenomenon of a human brain investigating for itself how that particular human brain works. These peculiar or abnormal manifestations of mental phenomena excite a good deal of misunderstanding, interest, and superstition.

The first and most essential fundamental fact underneath this whole question is the power of a suggestion, be it from an individual or contact with things, to set in motion a certain line of muscular activity by action through the nervous system. I use the following illustration to classes when dealing with this subject in its practical aspect, particularly with reference to the control and the care of children during the first visit to our offices. It is a homely suggestion, but I would like to give it to you now. No doubt you have all had similar experiences.

Not very long ago I saw upon a bill-board a statement in white letters on a blue ground, to the effect, "Uneeda Biscuit," and the first effect of it upon me was for me to say mentally, "Not much; I do not need a biscuit, and I will not permit anybody to tell me that I need a biscuit, when I know whether I need it or not." At every bill-board I passed was the legend, "Uneeda Biscuit." I grew more and more irritable about the thing, and I made up my mind that I would never buy one and would have nothing to do with the man who had the impertinence to urge the suggestion. I kept in that mental attitude for some time, and I said, "I will not read the thing." About a week afterwards the advertisement had changed somewhat and now read, "Do you know Uneeda Biscuit?" Well, I knew I did not, but, looking at it again, the miserable thing took a new aspect, and raised a doubt that I might be mistaken. It took some days for that to work out, and I thought the only way I could determine the question was to go and buy one, and see if I did need it. What was accomplished? Why, I had taken a part of my hard-earned wealth and gone to a shop and given it to a man in order to settle a point raised by a suggestion, and that is just what the advertisers aim at. I understand the firm has grown wealthy through the potentiality of that suggestion.

Let us take, for example, the child patient, or any subject upon which we wish to perform this experiment or get anything done. Let us say, for example, it is the extraction of a tooth or infliction of pain. The individual's mind is actuated by that idea,—I am to suffer pain,—and his whole muscular and mental attitude is dominated by the force of that suggestion. It is the business, then, of the operator to annul or get rid of that mental idea; and the object in hypnotic suggestion is to devise a means to annul that controlling idea and substitute therefor some other idea. That is as I understand it. We have to get rid of that muscular and mental *status quo* of that patient by some device or other, and then we can insert, as it were, this new suggestion and take advantage of that power or force. While not speaking dogmatically, I do not regard hypnotism as a power or force. It has no potentiality in itself. Whatever effect is produced is original within the subject hypnotized. I do not believe a force, an aura, or potentiality goes out from the operator. I am not ready to accept it, but I do not assert that it is not true.

Illustrative of the idea of elimination of fear referred to by the essayist and by Dr. Whitslar, I have in mind a very interesting case. We had in the class at college a student from California, who was assigned a day for extractions. Not a single patient appeared until late in the afternoon, when a woman put in an appearance and demanded an extraction with gas of some four or five teeth. As there was no demonstrator at that time, and no gas apparatus available, he explained to her that they did not give gas at that time. She was about to leave, and to avoid losing his opportunity he said, "I will give you electricity." She consented; so from his locker he brought two spirit lamps, and placing one in each of her hands he directed her to hold them very firmly and to open her mouth, which she did. He took out five or six teeth while I stood by as much hypnotized as the patient. I expected momentarily to see the patient leave the chair in indignation, but she sat with a perfectly placid expression while he removed one tooth after another. When he was through, she said that she had felt very little pain, and thanked him cordially. Not all persons are as impressionable as that patient.

*Dr. James Truman.*—I cannot believe that there is no fluid, or power, passing from the operator. I have performed this a great many times, and I am confident it is more than mere suggestion when you get to the hypnotic stage.

Dr. Schamberg speaks of post-hypnotic suggestion. Now, I think there is a vast field there, of very great value if it were only properly applied. We have a great many persons who are in the bad habit of using different drugs. Others smoke too much. To illustrate this: A powerful hypnotist had a friend, a gentleman, who was an hypnotic subject and a very great smoker, and while he was in the hypnotic state, she made him promise that he would not smoke for two weeks, while he, of course, knew nothing about it. When he came out of the sleep he went home and to his store. The next day he wanted to take a smoke. He could not smoke and did not. Every time he took the cigar there would be that opposition. I cannot see how mere suggestion would carry that far. At the end of two weeks he took a cigar and smoked it. On another occasion he started from the house to go home. When he had reached three blocks away she willed that he should come back. He did not know anything about that, could not possibly have known it. But he could not take a step forward. He tried, but could not move, and had to turn back to the house.

Now, these are a few facts out of many hundreds that might be related indicating a power that we are more or less ignorant of, but it seems that there is some mental power that can be carried a very great distance. I have seen people put to sleep by an operator some distance off in the same room without further manipulation than the pretence of magnetizing a glass of water, and can vouch for the phenomenon, as the subject was one of my family.

*Dr. McCullough.*—Some four years ago a patient was introduced to me with the understanding that she was to be treated with great care. From her appearance it seemed that she was suffering with cardiac debility or anæmia. At the first visit she fainted three times within fifteen minutes without my having said anything to her at all. After recovery from these faints I assured her that I was only going to examine her teeth and that I did not have time to do anything else. The next time she fainted, but I did some slight operation. The third time she did not faint, but jumped out of the chair and started to walk around the office. Fearing she might faint, I walked around back of her, for I was fearful lest she might fall. When I discovered that this young lady fainted so readily, I thought she might be induced to faint. Therefore, I prepared on a special occasion to extract a very bad tooth. As soon as everything was ready I told her I was going to extract the tooth. I presented the forceps and she fainted. Fortunately her jaw fell down and I extracted the tooth, but before the tooth was out of her mouth she came to, and afterwards said she absolutely felt no pain. I have not seen this young lady for two years, and on receipt of the card announcing the subject for this evening I naturally recalled my experience. The next day a messenger came from her, asking for an appointment.

*Dr. Kirk.*—I think I was very explicit when I said I would not dogmatically assert that it was not so, but I have never had any experience in my life to lead me to believe that there was such a thing as thought transferrence. I am sceptical about the possibility of explanation on the basis we have just heard related. I think if each one of us would make a record of the remarkable coincidences that happen in one's life, that are pure coincidences, that they would have a definite scientific value, as apparent causes of telepathy. But the power of suggestion and of post-hypnotic suggestion are both, it seems to me, perfectly explainable upon the basis of the very great potentiality of an idea or of a suggestion in originating ideas.

*Dr. James Truman.*—How would that bring about unconsciousness?

*Dr. Kirk.*—Consciousness, I believe, is an active condition of the cerebral cortex. As soon as we obliterate functional activity, we must have sleep. That would be a negative condition, being opposed to consciousness, and just as in paralysis there is obliteration of the actual functionating of the cerebral cortex, we have a tendency towards sleep.

*Dr. McCullough.*—I have read the physiological explanation of sleep. I understand that the phenomenon of sleep is explained as anæmia of the brain. Of that I am not sure, but it seems to me that that point was made in an argument, wherein it was proved that hypnotism would produce artificially an anæmic condition of the brain. A shining object before the eyes, upon which the patient has concentrated his sight, has the effect of producing this anæmia, though the fact is that never was our mind concentrated upon any one object to the total exclusion of everything else. Concentration like this has the effect of producing an anæmia of the brain, which in its weak condition is exposed to the influence of the operator.

*Dr. Neall.*—I have nothing more to say except to state how Mesmer proceeded to hypnotize his patients. It was to keep them in a dimly lighted room. Sometimes the hands were touched; at other times they were bound by a silken cord. He would enter the room in a fantastic garb, and would touch one person and then pass around to another. Men would have palpitation of the heart, and women would shriek. As to carrying this to the *true* hypnotic stage, it, of course, would not be the proper thing at all without a third person present. I have not in my office carried it to that period, but simply to that hypnotic *suggestion* in which patients seem to yield to the control of the operator, and have abandoned the idea of fear of pain until the operation is completed.

It can be practised if one makes up his mind to study the art, but not upon everybody. If you want to get a person into a state where you can control them mightily, generally suspend some bright article about eight or ten inches above the eyes or forehead, so that, in looking at it, the eyes are raised and also the eyelids, and if a person is to be impressed at all, he will come into that condition in from ten to twenty minutes, and be unable to control the eyelids or part the clasped hands if told he cannot. As a boy I used to watch my grandfather do this. He was one of the operators who extracted teeth on a public platform when the subject was hypnotized after this manner. I have seen the same things done at a private seance. I should imagine that any person with strength of mind and determination could be able to do this with a subject that can be impressed.

I would avoid taking into the office Class 4, unless somebody were present. I do not operate without having a lady attendant, so there would be no danger of scandal arising from it.

OTTO E. INGLIS,
*Editor Academy of Stomatology.*

